# Nationwide Seroprevalence of Leptospirosis among Young Thai Men, 2007–2008

**DOI:** 10.4269/ajtmh.17-0163

**Published:** 2017-10-02

**Authors:** Siriphan Gonwong, Thippawan Chuenchitra, Patchariya Khantapura, Dilara Islam, Nattaya Ruamsap, Brett E. Swierczewski, Carl J. Mason

**Affiliations:** Department of Enteric Diseases, and Division of Research, Armed Forces Research Institute of Medical Sciences, Bangkok, Thailand

## Abstract

Leptospirosis, a global neglected zoonotic disease, is an important public health problem in Thailand. Nonspecific symptoms, lack of laboratory confirmation, and underreporting contribute to its neglected disease status. To better understand the distribution of leptospirosis exposure in Thailand, a retrospective leptospirosis seroprevalence study was conducted on repository serum specimens obtained from young Thai men entering the Royal Thai Army during 2007–2008. The overall nationwide leptospirosis IgG seroprevalence among these young Thai men was 28% (95% confidence interval = 26–30%) and the range by province was 10–52% confirming leptospirosis as an endemic disease throughout Thailand. Seroprevalence was highest in individuals with the lowest education from rural areas, and higher seroprevalence was found in the north and south regions contrary to current morbidity reports. Improvement in reporting and surveillance as well as better access to leptospirosis diagnostics will increase leptospirosis awareness and detection and enable more effective public health interventions.

Leptospirosis is a global zoonotic disease caused by a Gram-negative spirochete belonging to the genus *Leptospira*. Humans and animals are infected with *Leptospira* through direct contact with the urine of an infected animal or indirectly through environmental contamination such as soil and water. *Leptospira* organisms enter through mucous membranes (mouth, nose, and eyes) or through cuts or abrasions on the skin. Clinical signs of leptospirosis vary from a mild, flu-like, self-limited febrile illness to a fulminant life-threatening illness with multiorgan system complications leading to death.^1–3^ Leptospirosis is an emerging global disease with an estimated 1.03 million cases and 58,900 deaths in 2015 and is most prevalent in developing countries in tropical regions.^2^ Young adult males (20–29 years of age) are at high risk for leptospirosis because of their occupational activities in these countries.^2,3^

Leptospirosis is an emerging public health problem in Thailand, with the first confirmed case reported in 1942. The annual case reports were low before 1996, with approximately 200 cases reported annually mainly from the central and south regions.^4,5^ Leptospirosis is listed as a notifiable disease with voluntary reporting but not on the list of statutory notifiable diseases in Thailand.^4^ Since 1996, leptospirosis case reports dramatically increased with outbreaks reported in the northeast region. A major outbreak of leptospirosis occurred in 2000 with 14,285 cases and 362 deaths reported nationwide with an incidence and case fatality rate at 23.7 per 100,000 person-years and 2.7%, respectively.^5^ Case reports and morbidity of leptospirosis in Thailand have declined since 2001 ranging from 2,251 to 10,217 cases yearly and 16.45 to 3.47 reported cases per 100,000 person-years from 2001 to 2014.^6^ Seroprevalence data of leptospirosis in the Thai general population are limited. A nationwide leptospirosis seroprevalence study (using agglutination tests) was conducted in 1966 on nonfebrile adult patients in hospitals across Thailand.^7^ A more recent leptospirosis seroprevalence study in Thailand may provide disease burden information for disease control.

The microscopic agglutination test (MAT) has been the gold standard method for diagnosis of human leptospirosis. As the MAT requires maintenance of serovars of live *Leptospira* strains as well as expertise in reading and interpreting the results, enzyme-linked immunosorbent assays (ELISAs) for leptospirosis have been developed and used in multiple studies.^8,9^ The detection of IgG antibodies against *Leptospira interrogans*, the major pathogenic strain in Thailand, was performed by ELISA in this study.^10^ Repository serum specimens from Royal Thai Army (RTA) recruits were used to determine leptospirosis IgG seroprevalence reflecting nationwide exposure and distribution of leptospirosis in young Thai men.

A stratified random sample of 7,760 sera were selected from 121,370 repository serum specimens obtained under informed consent with permission for future studies as part of a previous HIV-1 surveillance study among young Thai men of age 18–30 years entering the RTA during 2007–2008. Thailand is divided administratively into 76 provinces including the capital, Bangkok. Provinces are subdivided into districts. The men entering service comprise approximately 10% of all young men at the district level in Thailand.^11^ Sample sizes were calculated to detect a seroprevalence of approximately 50% in each province to within 10% of the true value with 95% confidence. This study was approved by the Institutional Review Board, Royal Thai Army Medical Department, Bangkok, Thailand, and approved as exempt by the Human Subjects Protection Branch, Walter Reed Army Institute of Research, Silver Spring, MD, USA.

IgG antibody against *L. interrogans* was measured using a modified *Leptospira* IgM ELISA kit (Panbio Ltd., Brisbane, Australia). Procedures were performed according to the manufacturer’s instructions except horseradish peroxidase (HRP)-conjugated goat anti-human IgG (H + L) (Kirkegaard & Perry Laboratories, Gaithersburg, MA) was used in lieu of HRP-conjugated sheep anti-human IgM.^9^ The cut-off was set at 2.3 SD above the mean optical density of 61 negative control sera.^8^ Positive and negative controls (Panbio) were included in duplicate on each assay plate.

Associations between demographic characteristics and leptospirosis seroprevalence results were tested by χ^2^ two-tailed test. Correlation of leptospirosis seroprevalence with land use data was analyzed using Spearman’s correlation coefficient test. Statistical analyses were tested using SPSS version 12 (SPSS, Chicago, IL); a *P* value < 0.05 was considered statistically significant.

The study population was mostly 21 years of age, unmarried, and a junior high school graduate living in a rural area (Table 1). The sample size per province ranged from 69 to 130 samples. The overall leptospirosis seroprevalence was 28% (95% confidence interval = 26–30%) and seroprevalence by province ranged from 10% to 52% suggesting endemicity throughout Thailand. The leptospirosis IgG seroprevalence was highest in the north and south regions at 33%. A choropleth map of leptospirosis seroprevalence by residential province was generated with ArcView 8.3 (ESRI, Redlands, CA) (Figure 1).^12^ Our findings are consistent with the previous nationwide leptospirosis seroprevalence study conducted in 1966 that showed an overall nationwide seroprevalence of 27% in the Thai general population with higher seroprevalence detected in the south region.^7^ However, the comparisons are limited by differences in study design, population investigated, and method of antibody detection.

**Table 1:** Leptospirosis seroprevalence in association with demographic variables in young Thai men, 2007–2008

Demographic characteristics	Study subjects no. (%)*	Leptospirosis IgG seroprevalence, % (95% CI)†
Total	7,760 (100)	28 (26–30)
Age group (years)‡		
18–20	1,164 (15)	20 (18–22)
21	5,359 (70)	25 (24–26)
22–30	1,150 (15)	23 (21–26)
Education level‡		
Primary school and less	2,121 (27)	28 (26–30)
Middle school	2,641 (34)	24 (23–26)
Senior high school and vocational	1,920 (25)	22 (20–23)
Diploma, high vocational and bachelor’s degree	1,061 (14)	18 (16–21)
Marital status		
Single	6,067 (80)	24 (23–25)
Married	1,509 (20)	24 (21–26)
Residential area‡		
Urban	2,503 (39)	19 (17–20)
Rural	3,896 (61)	26 (24–27)
Region of residence‡		
Central	2,626 (34)	22 (19–26)
North	1,738 (22)	33 (28–38)
Northeast	2,004 (26)	27 (24–30)
South	1,392 (18)	33 (29–38)

* Number in each demographic characteristic does not add to the total number of study subjects because of missing data; the number of study subjects with data missing for age group, marital status, education level, and residential area are 87, 184, 17, and 1,361, respectively.

† CI = confidence interval.

‡ χ^2^ test statistically significant (Pearson’s χ^2^, two-sided, *P* < 0.05).

**Figure 1.**
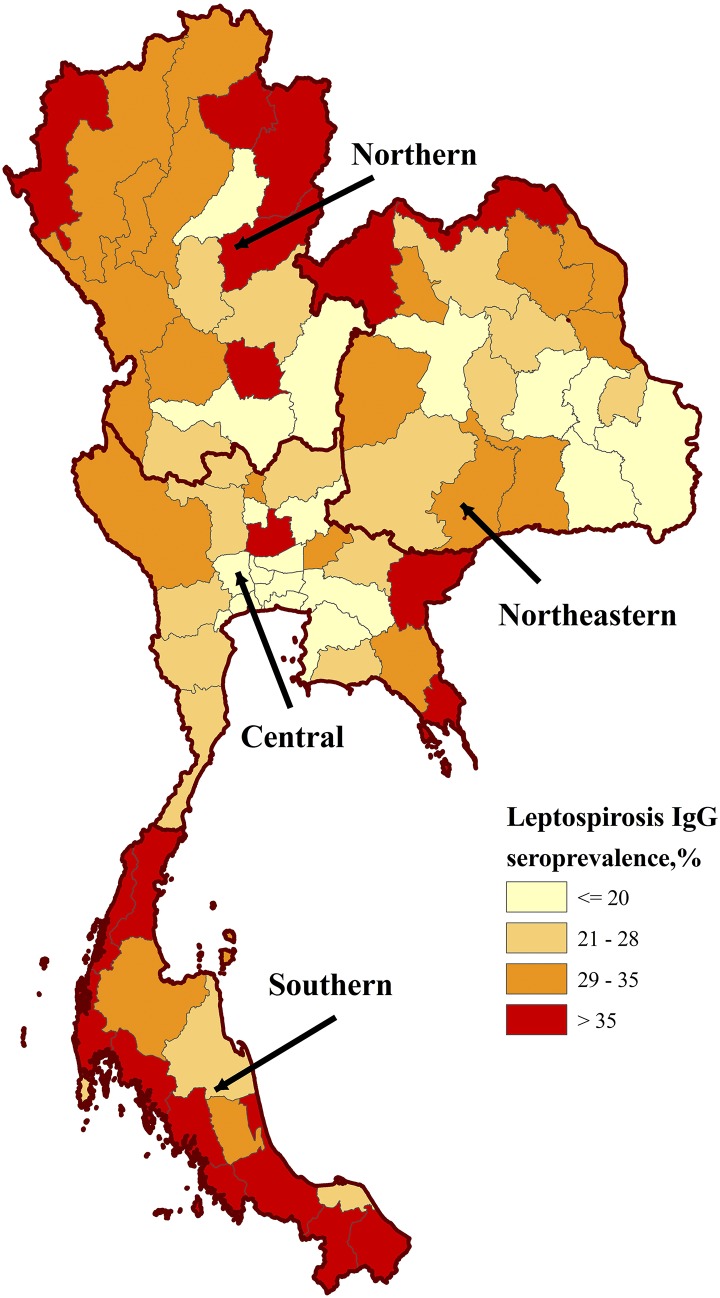
Choropleth map of leptospirosis seroprevalence in young Thai men, 2007–2008. Prevalence is stratified by color and location determined by reported residence province during the 2 years before Royal Thai Army enlistment. Thailand shapefile in the public domain.^12^

Using univariate analysis, leptospirosis IgG seropositivity was significantly associated with older age groups, lower education levels, residence in rural areas, and region of residence (Table 1). The findings of increased seroprevalence in our study among males aged 21 years and above is consistent with previous findings that adult males aged 20–29 years are at higher risk of acquiring leptospirosis.^2^

Previous studies have shown that leptospirosis is an occupational disease mainly affecting rural subsistence farmers. The majority of leptospirosis cases reported in Thailand from 1995 to 2003 were farmers (71.5–83.5%).^2,5^ Occupational data were not available in this study; however, the National Statistical Office reported that the majority (75%) of Thai males who worked in agriculture and fisheries had primary school or less education.^13^ Our finding of increased leptospirosis seroprevalence in primary school and less educated, rural residents may be related to the agriculture or fisheries industries.

A previous study in Southeast Asia showed that *L. interrogans* in rodents was linked to humid habitats such as forested areas.^14^ Land use data from the Land Development Department, Ministry of Agriculture and Cooperatives, Thailand, are shown in Table 2.^15^ An ascending trend in forested land and a descending trend in urban and built-up land was correlated with increasing leptospirosis seroprevalence (Supplemental Figure 1) (Spearman’s rho = 0.47 and −0.65, respectively, *P* < 0.001 for both). This finding corresponds with the higher seroprevalence found in individuals living in rural (26%) as opposed to urban (19%) areas (Table 1). The finding of higher leptospirosis seroprevalence in provinces with higher percentages of forested land supports previous studies.^14,16,17^ In addition, agriculture and water have been reported as factors related to leptospirosis transmission.^18^ However, no association with agriculture and water was observed in this study because of limitations in agriculture land and water body data resolution.

**Table 2:** Percentage of land use data and morbidity rate in each magnitude of leptospirosis IgG seroprevalence in young Thai men, 2007–2008

Leptospirosis IgG	Median % land use data* (Q1, Q3)				Median of morbidity rate 2005–2008† (per 100,000) (Q1, Q3)
Seroprevalence‡	Urban and built-up land§	Forest land‖	Agricultural land	Water body	
Low (≤ 20%)	7.5 (5.2, 16.8)	8.2 (0.1, 16.4)	65.7 (58.1, 75.0)	2.7 (2.2, 3.8)	1.1 (0.1, 3.7)
Medium (21–28%)	5.6 (4.6, 6.2)	21.6 (12.2, 37.5)	64.6 (46.5, 74.5)	2.2 (1.4, 3.4)	2.3 (0.5, 6.3)
High (29–35%)	3.6 (2.6, 4.8)	31.7 (22.4, 63.0)	57.6 (28.0, 66.4)	2.3 (1.5, 4.3)	2.7 (0.9, 8.8)
Very high (> 35%)	2.9 (2.3, 3.9)	33.8 (23.4, 57.0)	59.1 (35.5, 68.9)	2.3 (1.3, 4.5)	4.6 (1.3, 10.8)

* Land use data from Land Development Department, The Ministry of Agriculture and Cooperatives, Thailand. The land data were assembled from geographic information system and field site survey.^15^

† The Annual Epidemiology Surveillance Report from the Ministry of Public Health, Thailand.^6^

‡ *N* = 19 provinces/group.

§ Spearman’s rho = −0.65, two-sided, *P* < 0.001.

‖ Spearman’s rho = 0.47, two-sided, *P* < 0.001.

Ambiguity of morbidity and mortality rates is a major reason leptospirosis is considered a neglected tropical disease. As a notifiable disease with voluntary reporting, the highest reported morbidity is in the northeast region.^4,6^ This is contrary to our findings of higher seroprevalence in the north and south regions supporting that seroprevalence in Thailand is inconsistent with reported incidence. For example, leptospirosis seroprevalence did not correspond to morbidity reports in provinces such as Mae Hong Son, Sa Kaeo, and Satun where seroprevalence was high at 52%, 45%, and 44%, respectively, but reported morbidity was unexpectedly low at 2.8, 1.5, and 2.8 cases per 100,000 person-years in 2008, respectively.^6^ Underestimation of leptospirosis burden results from mild or subclinical cases, misdiagnosis due to the inability to distinguish leptospirosis from other endemic febrile diseases and inaccessible or inadequate laboratory capability/detection.^4,5^ Previous studies reported poor clinical diagnosis of leptospirosis in Thailand with 0% detected in the south region to 50% in northeast region.^5^

Our study results are limited by study design, population investigated (male Thai recruits), and sample collection from a single period. In Thailand, the reported incidence of leptospirosis is higher in males than females, with a case ratio of 3.6:1 and 3.9:1 in 2007 and 2008, respectively, with an incidence peak reported at 55–64 years of age.^19,20^ Our nationwide retrospective leptospirosis seroprevalence study in young Thai male recruits reflect the exposure of leptospirosis in the general young male adult population as well as the overall geographic distribution of human leptospirosis in Thailand. For future seroprevalence studies, it will be important to collect samples from a general population with varying genders and age groups.

In summary, leptospirosis seroprevalence in all provinces of Thailand confirms endemicity. Unlike studies focusing on case reports, our seroprevalence data detected nationwide *Leptospira* exposure in young Thai males. Leptospirosis seroprevalence was found to be associated with individuals aged 21 years and older with a primary school or less education level living in rural areas. Higher seroprevalence was found in the north and south regions contrary to reported morbidity and potentially associated with environments such as forested and rural areas. Improvement in reporting and surveillance as well as better access to leptospirosis diagnostics will increase leptospirosis awareness and enable more effective public health interventions.

## Supplementary Material

Supplemental Figure.
