# Supplementary material for: Nationwide Seroprevalence of Leptospirosis among Young Thai Men, 2007–2008
Source: Am J Trop Med Hyg. 2017 Oct 2;97(6):1682–5. doi: 10.4269/ajtmh.17-0163 (PMC5805037; doi:10.4269/ajtmh.17-0163)
Supplement: Supplementary file 1 [file tpmd170163.SD1.pdf]

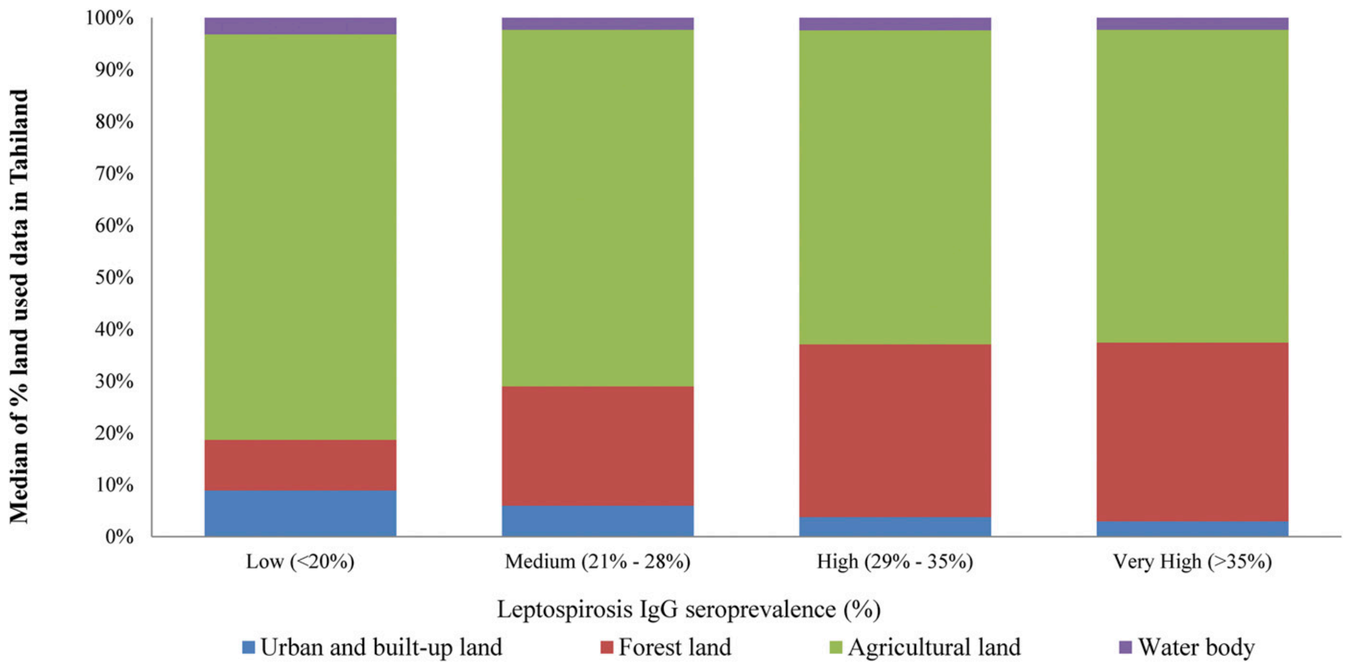

Supplemental Figure 1. Percentage of land use data grouped by magnitude of leptospirosis IgG seroprevalence in young Thai men, 2007–2008.
